# Securing schools, protecting minds: a scoping review of limited evidence for weapon carriage prevention in K-12 schools

**DOI:** 10.1080/28324765.2025.2603596

**Published:** 2025-12-16

**Authors:** Sarah M. Stilwell, Paulina Guzmán M., Justin Heinze, Marc Zimmerman

**Affiliations:** a University of Michigan School of Public Health, Department of Health Behavior and Health, Equity, Ann Arbor, Michigan, USA; b University of Michigan Institute for Firearm Injury Prevention, Ann Arbor, Michigan, USA; c Universidad San Sebastián, Facultad de Educación, Santiago, Chile

**Keywords:** Weapon carriage, school violence, violence prevention, intervention, scoping review

## Abstract

Rising rates of weapon carriage in U.S. K–12 schools threaten the safety and well-being of students, educators, and staff. Despite widespread use of security measures such as metal detectors and law enforcement presence, evidence supporting their effectiveness remains mixed. This scoping review analyzes empirical studies from 2005 to 2025 that evaluate interventions aimed at reducing weapon carriage in U.S. schools. Only two studies met inclusion criteria: one assessing the impact of random mandatory metal detector searches on weapon carriage and student safety perceptions, and another evaluating the Say Something Anonymous Reporting System (SS-ARS) for its potential to prevent firearm-related incidents. While both interventions showed limited positive outcomes, the overall evidence base is weak, and long-term effectiveness is unclear. Moreover, ‘target hardening’ strategies like metal detectors may negatively affect student perceptions of safety, particularly among students of color. Findings highlight the need for more holistic, evidence-based approaches that go beyond physical security. Interventions that emphasize social and emotional learning, mental health support, and positive school climate may better address the root causes of weapon carriage. Future research should focus on evaluating integrated strategies that balance physical safety with psychological well-being to create more supportive and secure school environments.

School safety is a significant concern for parents, policymakers, and the public, as incidents of school violence generate fear and anxiety among students and families (Cohen, 2021; Mayer et al., 2021). Firearms are now the leading cause of death among U.S. children and adolescents, and concerns about school violence are widespread, and three out of every four adolescents in the United States worry about being a victim of school gun violence (Kirkland et al., 2026; Lee et al., 2022). Reflecting on these fears, the percentage of students reporting being threatened or injured with a weapon (e.g. firearms, knives) on school property is rising, increasing from 7% in 2021 to 9% in 2023, according to the Youth Risk Behaviour Survey (CDC, 2021; 2023). These trends underscore the pressing need for effective prevention strategies, as even isolated incidents of weapon-related violence can have far-reaching effects on school climate and student well-being.

School-based weapon carriage is associated with a range of adverse outcomes, including increased risk of physical violence, psychological distress, and reduced perceptions of safety among students (Brennan & Moore, 2009; Forrest et al., 2000; Lowry et al., 2023). Furthermore, exposure to violence or the perceived potential for violence contributes to heightened stress, anxiety, depression, and feelings of unsafety among youth, which in turn can affect academic performance, social functioning, and overall well-being (Ferrara et al., 2019; Kim et al., 2020). As such, addressing school safety and weapon carriage is not only a matter of physical protection, but also a critical issue for safeguarding student well-being.

National data indicate that approximately 6% of students in grades 9–12 reported carrying a weapon on school property within the past 30 days, a number that continues to rise (NCES, 2022). Researchers have estimated that 3-6% of students carry weapons to school, which means with over 1.5 million high school students in the U.S., *tens of thousands* of weapons are brought into schools each year (NCES, 2022). This behaviour not only compromises the physical safety of students, teachers, and staff but also has serious implications for mental health. Carrying weapons in schools has been consistently linked to adverse effects on the mental health of both students who carry weapons and those who, without doing so, perceive a persistent threat of violence in their school environment.

For students who carry weapons, several studies have documented a significant increase in symptoms of anxiety, depression, aggression, impulsivity, post-traumatic stress, and even antisocial traits (Bhandari, 2014; Johnson et al., 2019). For instance, students who report carrying a weapon on school property have more than double the odds of attempting suicide compared to their peers who do not carry weapons (Baiden et al., 2019). Moreover, a bidirectional relationship has been identified between emotional distress and gun ownership: on the one hand, students may resort to carrying guns as a strategy for self-protection or power assertion; on the other hand, such behaviour can intensify stress and exacerbate pre-existing psychological problems (Watts et al., 2019; Wilcox et al., 2006). In particular, one study found that handgun carrying was associated with increased fear of victimisation, suggesting that weapon possession may reinforce, rather than reduce, feelings of vulnerability (Wilcox et al., 2006).

The detrimental effects are not limited to those who carry weapons. Students who attend schools where weapons are prevalent often experience heightened psychological distress, fear, and feelings of insecurity, even if they do not personally carry weapons (Borowsky et al., 2004). This pervasive sense of threat can erode students’ overall well-being, diminish their sense of belonging, and undermine academic engagement.

These dynamics underscore the importance of interventions that address both the psychological antecedents and consequences of weapon carrying, targeting perceptions of safety as well as underlying emotional distress. Given the seriousness of these trends and the limited scholarly attention to comprehensive, evidence-based strategies for preventing the carriage of weapons in schools, this paper seeks to review existing research to identify effective interventions.

## The effects of weapon violence on school safety

The factors influencing weapon carriage in schools are multifaceted, involving individual, social, and environmental determinants. Individual determinants may include personal experiences with victimisation, a perceived need for self-defence, and demographic risk factors such as prior suspensions or involvement in fights. Studies have shown that adolescents with histories of violent behaviour, multiple suspensions, or fear of being victimised are more likely to carry weapons to school (Bailey et al., 1997; Khoury-Kassabri et al., 2007; Moore, 2016).

Social determinants, such as peer influence and school connectedness, also play a critical role. Stoddart and Britto (2024), for example, found that school connectedness and social support play a critical protective role in reducing both firearm-carrying and weapon-related violence in schools​. Students who feel more connected to their schools and receive support from peers, teachers, and parents are significantly less likely to carry firearms. Conversely, weak social ties and exposure to violence increase the likelihood of weapon carriage among adolescents. Schools with low levels of student engagement and weak adult relationships show higher rates of weapon carriage, while stronger bonds with parents, teachers, and peers reduce the risk (Brank et al., 2007; Cunningham et al., 2022; Stoddart & Britto, 2024). Peer risk behaviour and pro-gun socialisation also significantly increase the likelihood of students bringing weapons to school (Beachy & Liang, 2024).

Finally, environmental determinants include neighbourhood safety and exposure to community violence. Adolescents living in high-crime or economically disadvantaged neighbourhoods are more inclined to bring weapons to school for self-protection. Studies have found that schools with high concentrations of low-socioeconomic status students exhibit greater incidences of weapon carriage (Wilcox & Clayton, 2001), and that fear and victimisation related to unsafe neighbourhoods are strongly predictive of weapon carrying (Khoury-Kassabri et al., 2007). For instance, Baiden et al. (2024) found that adolescents exposed to neighbourhood violence are significantly more likely to carry firearms, indicating a behavioural response rooted in perceived insecurity and chronic stress.

Trends in school-based weapon carriage indicate that certain groups of adolescents are at elevated risk. Most adolescents who carry firearms do so intermittently and typically for self-defence or protection. Carriage is notably more common among youth with prior experiences of victimisation, especially those who are both victims and offenders (Holt & Gini, 2017). Students who value academic success and report high school engagement, including putting effort into schoolwork or believing grades are important, tend to be less likely to carry weapons (McCuddy et al., 2024). Rates of firearm carriage are higher among adolescent Black males, particularly those involved with the criminal justice system, reflecting broader systemic inequalities and increased exposure to violence. Additional risk factors include substance use, mental health challenges (e.g. stress, PTSD), access to firearms, prior involvement in violence, and association with delinquent peer groups (Oliphant et al., 2019). For instance, recent findings from Dalve et al. (2023) further underscore that youth who carry handguns to school—especially in rural areas—exhibit significantly higher levels of individual, peer, school, and community risk factors, such as prior violent behaviour, peer delinquency, and feeling unsafe at school, compared to their peers who either do not carry or carry only outside of school contexts.

Furthermore, the presence or threat of firearm violence in schools has serious consequences for academic outcomes. Even in the absence of actual violence, fear of gun-related incidents can result in chronic stress, hypervigilance, anxiety, and depressive symptoms among students (Riehm et al., 2021). These psychological burdens can interfere with concentration, memory, and motivation, ultimately hindering academic performance. The fear of school-based gun violence can lead to chronic stress and anxiety in students, making it difficult to focus on schoolwork or participate in school activities (Riehm et al., 2021). Addressing the problem of weapon carriage in schools is critical for creating safe and secure learning environments for everyone.

## Effectiveness of current prevention strategies

Current literature proposes a comprehensive, multilevel approach to addressing school violence, combining primary, secondary, and tertiary prevention strategies (Lester et al., 2017; Mayer et al., 2021; Rajan et al., 2022;). These strategies seek not only to respond to violent incidents but also to prevent their occurrence by addressing their underlying causes and subsequent effects.

Primary prevention interventions focus on improving structural conditions in schools and communities before violence occurs. Rajan et al. (2022) emphasise the importance of community investments, including improvements to public lighting, access to affordable housing, and expansion of public libraries. These measures not only reduce armed violence but also promote overall well-being, particularly in communities with high exposure to violence.

Within the school environment, promoting a positive climate is essential. Rajan et al. (2022) argue that respectful teacher-student relationships, clear and consistent rules, and opportunities for student participation contribute to reducing the risk of violence. Mayer et al. (2021) agree on the importance of strengthening the school climate as a central focus of improvement efforts, emphasising that integrated models such as SEL (Social Emotional Learning) and SWPBIS (School-Wide Positive Behavioural Interventions and Supports) should be coordinated to respond to the psychosocial diversity of students. Another critical component of primary prevention is controlling access to firearms. Rajan et al. (2022) emphasise that more restrictive state laws, such as bans on assault weapons or raising the minimum age for purchasing semi-automatic weapons, are associated with lower rates of school shootings.

Despite the emphasis on structural and relational approaches, many schools have also adopted more security-oriented strategies in an attempt to reduce firearm possession and respond to immediate threats. Efforts to address firearm carriage in schools have led to the implementation of multiple safety strategies, including the presence of law enforcement officers, the use of metal detectors, and the adoption of anonymous reporting systems. While these measures seek to prevent the entry of firearms and deter gun-related violence, the evidence on their effectiveness in reducing firearm carriage specifically is limited and inconclusive (Perumean-Chaney & Sutton, 2013; Schildkraut & Grogan, 2019). Additionally, technological advancements have led to the increased use of strategies such as surveillance cameras and metal detectors, particularly in urban school districts (Kupchik & Ellis, 2008; Servoss & Finn, 2014). Researchers have reported emerging evidence that the use of metal detectors may not be a positive approach to reducing school violence and may have detrimental effects on student perceptions of school safety, particularly for youth of colour (Gastic & Johnson, 2015). The *Say Something Anonymous Reporting System (SS-ARS)*, for example, has been utilised in more than 50% of the U.S. K-12 schools, helping identify and intervene in potentially violent situations​ (Thulin et al., 2024). Over a four-year period, this system reportedly prevented 38 acts of school violence, including incidents where weapons were recovered on school grounds. However, questions remain about the long-term efficacy of such interventions, particularly their ability to address the root causes of firearm-related violence in schools, and raise concerns about what additional measures are needed to prevent weapons from entering schools in the first place.Another critical aspect of firearm possession in schools is the role of school policies and law enforcement responses. The *Gun-Free Schools Act (GFSA)* requires mandatory expulsion for students who bring firearms to school, yet enforcement and effectiveness of such policies vary by jurisdiction​ (Pulavarthi et al., 2024). Data from the 2020–2021 school year indicate a decline in reported firearm incidents, largely attributed to COVID-19 school closures (Jones et al., 2023). However, weapons still persist in schools. More recent national and state-level reports show that this decline was short-lived. Following school reopenings, firearm-related incidents, threats, and weapon detections have rebounded substantially (CDC, 2023; NCES, 2022). This post-COVID resurgence underscores how quickly weapon carriage risk can return when protective structures and preventing supports are not sufficiently maintained. It also highlights the urgent need for comprehensive, evidence-based strategies that move beyond punitive measures to address the underlying social and psychological factors contributing to school violence.

Despite the urgency of this issue, there is a significant gap in evidence-based strategies designed to address weapon carriage in schools. This gap highlights the complexity of creating effective solutions to ensure safe learning environments. Although interventions such as metal detectors and reporting systems are in use, the lack of definitive evidence supporting their efficacy leaves open questions about how to best prevent weapons from entering school environments. Given the lack of consolidated findings and the need for informed policy and practice, a scoping review is warranted to synthesise existing knowledge on strategies that seek to prevent, reduce, or eliminate the carriage of weapons, including firearms, by students or other unauthorised individuals in K–12 schools. These strategies may help prevent the underlying factors that lead students to bring weapons to school in the first place.

## Methods

### Search strategy

This scoping review identified and examined evidence-based interventions implemented in K–12 school settings to prevent or reduce weapon carriage on school grounds. The review followed a protocol informed by the Preferred Reporting Items for Systematic Reviews and Meta-Analyses Extension for Scoping Reviews (PRISMA-ScR) guidelines, however, the research protocol was not registered in a formal database (Moher et al., 2009; Tricco et al., 2018). The completed PRISMA-ScR checklist is shown in Appendix A (Tricco et al., 2018). In collaboration with a trained librarian, a comprehensive search strategy was developed and applied across the following six electronic databases: ERIC, PubMed, Scopus, PsycINFO, Sociological Abstracts, and Criminal Justice Abstracts.

We restricted the search to English-only studies and collected all database results published during the past 20 years (2005-2025) to capture how strategies have changed over the past two decades as school safety concerns and responses have continued to evolve. The search terms used addressed the main concepts of the search strategy: ‘weapons,’ ‘carriage,’ and ‘schools’ (see Appendix B for example search syntax). We intentionally omitted intervention-related terms from the Boolean search to avoid unduly restricting results, given the inconsistent terminology used to describe school-based interventions in the literature. Studies identified through this broad search were then manually screened to determine whether they met the criteria for school-based interventions. After the full-text review, we assessed the reference lists of included studies to identify any additional studies for inclusion, a process known as snowball sampling (Hiebl, 2023).

### Inclusion criteria

We included articles that reported on interventions developed and applied to prevent weapon carriage in a K-12 school. To be included in this review, articles had to 1) be published in English; 2) report on interventions in K-12 schools; 3) interventions conducted within the U.S.; and 4) be peer-reviewed articles. Following these inclusion criteria, we excluded articles that: 1) did not include or describe interventions to prevent weapon carriage in a K-12 school; 2) were not applied in schools K-12; 3) were not conducted within the U.S.; 4) were not published in English; and/or 5) were a review, editorial, opinion piece commentary or solely reported on implications for practice.

### Study selection

We used Covidence systematic review software (2024), a web-based collaboration software platform that streamlines the production of systematic and other literature reviews, to manage the screening and selection of articles. The coding team comprised three researchers: two senior scholars with expertise in violence prevention and research review methodology (Coders A and B), and one master’s-level researcher (Coder C) trained by the senior team. Coders A and B collaborated with the librarian to develop search terms and conduct database searches. Coder C joined at the screening stage and received structured training from the senior coders. All titles and abstracts were independently screened by two coders in a blinded manner to determine eligibility. Discrepancies were discussed and resolved through consensus during team meetings.

The same team members conducted full-text screening for relevant articles to determine whether each article should be included in the final sample. After reviewing the full texts, the research team developed criteria and procedures for what information to extract from the included studies. Two coding team members performed the initial data abstraction, and a third coding team member assessed the results for quality and accuracy. At each step, the research team met to discuss any discrepancies until a unanimous decision was reached regarding the inclusion or exclusion of a particular study from the final sample.

### Data abstraction

After determining the final studies for inclusion, the research team developed data abstraction procedures to collect key information from the full texts of the included articles. The data abstraction focused on several key sections, including: study year, methodology, participants, intervention descriptions and results. A primary reviewer conducted the initial data abstraction, which was subsequently reviewed for accuracy and quality by a secondary reviewer. Any discrepancies that arose were resolved through discussion and consensus among the team members.

## Results

**Figure 1.**
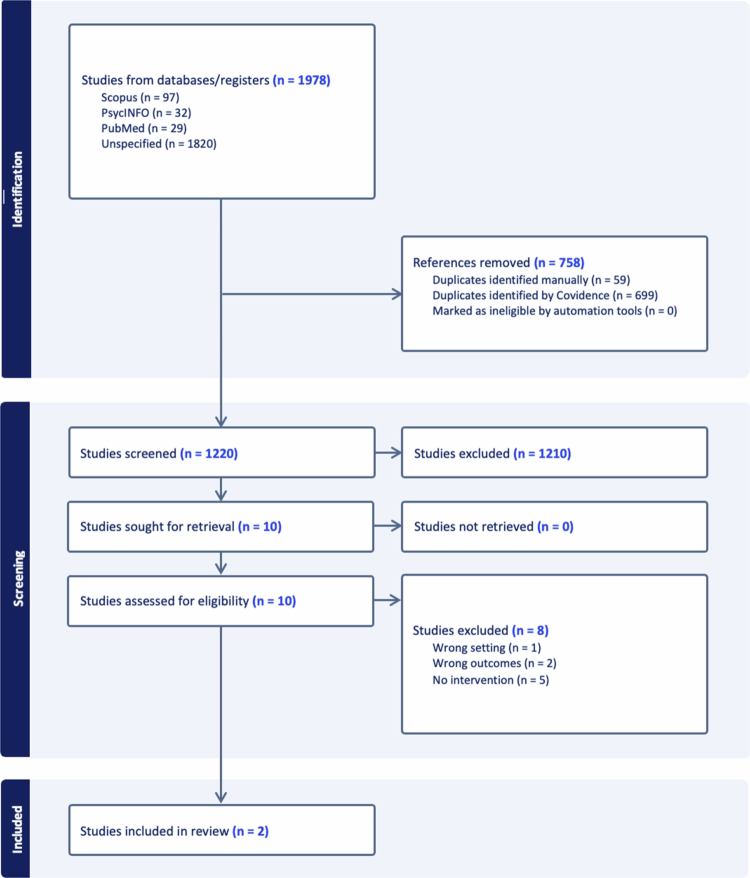
PRISMA flow diagram of included articles.

Our initial database search identified 1,220 unique articles (Figure 1). During our initial title/abstract screening, we excluded 1,210 articles based on the four inclusion/exclusion criteria described above (i.e. not intervention, K–12 schools, not published in English, or not considered peer-reviewed articles). In the second phase, all full-text articles (*N* = 10) were assessed for eligibility based on inclusion criteria. Of those, eight were excluded because they did not report on an intervention (*n* = 5), did not report on weapon carriage as an outcome (*n* = 2), or were not conducted in K–12 schools (*n* = 1). A final sample of two articles were included in this review.

### Studies included in the review

The first study was conducted during the 1998-1999 school year and sought to understand the relationship between random mandatory metal detector searches, reduction of contraband including weapons, and student perceptions of school safety (Bhatt & Davis, 2018). The researchers used pre (1993-1997) and post (1999-2007) Youth Risk and Behaviour Surveillance Survey data. They found that random searches were associated with students bringing fewer weapons to school. While this intervention did demonstrate some efficacy in the overall approach to reducing weapon carriage in schools, it did not have a comparison or control group, and was based on difference-in-differences estimates from only one district in one state. The second study, by Thulin et al. (2024), examined the effectiveness of an anonymous reporting system in preventing firearm-related incidents in schools. The researchers analysed four years (2019-2023) of data from the Say Something Anonymous Reporting System (SS-ARS), implemented across 103 school districts and 156 charter schools in a Southeastern state. They found that the system enabled 1039 confirmed mental health interventions, averted 38 acts of school violence (including recovered weapons on school grounds), and prevented six planned school attacks. While this intervention demonstrated promise in identifying and mitigating firearm-related threats in school settings, the study relied on retrospective administrative data, making it difficult to establish a direct causal link between anonymous reporting and reductions in school weapon carriage​. This study may help provide some evidence-informed guidance regarding best practices, but without an experimental design this study is limited.

## Discussion

Overall, while both studies suggest potential methods for reducing weapons in schools, the evidence remains limited, with no conclusive findings that directly demonstrate the effectiveness of these interventions in preventing weapons from entering school environments. Thus, the critical question arises: How can we create safe and secure learning environments in our schools? It is time to reconsider our approach. This gap underscores the need for further research into comprehensive, evidence-based strategies for preventing weapon carriage and enhancing school safety.

The limited number of studies identified through this review represent not only a lack of rigorous testing for particular strategies but a broader evidence gap across all forms of school- based prevention. This scarcity suggests that the field has yet to systematically evaluate whether the many safety initiatives already in place, ranging from physical security to social emotional learning, are effective in reducing the likelihood of weapon carriage.

The dearth of empirical evidence for prevention of weapon carriage in schools was surprising given so many schools across the U.S. have implemented physical detection systems (Perumean-Chaney & Sutton, 2013). Although metal detectors or other detection devices, including clear backpacks or other target hardening strategies seem to be intuitive, their utility lacks evidence (Hankin et al., 2011). While such mechanisms are used to secure other buildings (e.g. airports, courthouses), they may not make as practical for schools due to financial costs (Schildkraut & Grogan, 2019), trained personnel required to staff machines, and the unique context of many students entering the building during a short period of time (i.e. the minutes before the bell rings). In addition, a metal detector would have to be monitored throughout the day for visitors who enter the school during the entire school day. Interestingly, researchers have found that metal detectors and other target hardening strategies are associated with increased stress for students and undermine their feelings of safety (Gastic, 2011), but have not been tested in an evaluation comparing intervention strategies or using a comparison group design.

Despite the potential benefits of detecting weapons as they enter schools, these physical measures do not address the deeper social and emotional issues that may lead students to carry weapons in the first place. It may be argued that if a child brings a weapon to school, even if detected before it is discharged, that the system has failed to create a safe environment for learning. Rather, schools may need to consider more climate and social, and early detection strategies so students do not feel a need to carry a firearm or other weapon to school. Poor mental health, including conditions such as anxiety, depression, trauma-related stress, and suicidal ideation, is frequently associated with increased risk of violence and self-harm, both of which are factors linked to weapon carriage among adolescents. For instance, students who carry weapons are more than twice as likely to have attempted suicide compared to their peers (Baiden et al., 2019), and exposure to school violence can further intensify stress and anxiety (Riehm et al., 2021). Students may carry weapons as a form of self-protection, often rooted in fear and perceived threats, particularly when their emotional and psychological needs go unmet. As such, early detection of and services to improve mental health may be necessary components for weapon carriage prevention.

Some researchers argue that the use of physical security measures to prevent weapons from entering the schools encourages students to seek alternatives to introducing weapons into the school, such as bypassing the system (Hankin et al., 2011). Furthermore, metal detectors are costly to install and difficult to implement effectively and are often perceived as creating an uninviting environment for students, increasing fear, and reducing overall school climate (Gastic & Johnson, 2015). As such, a broader approach that incorporates early detection and social-environmental strategies, such as enhancing school climate and increasing mental health support, may be more effective in preventing weapon carriage (Stilwell et al., 2024).

Preventing weapon carriage in schools requires a multifaceted approach that may include physical security (target hardening) measures but also involves proactive social interventions. Strategies provide alternatives to physical hardening measures and move further up the prevention stream may be more effective. Stilwell et al. (2024) identify social and early detection environments of schools as vital strategies that seek to improve school social climate and reporting signs of distress can provide necessary support before a weapon enters the school (Messman et al., 2024; Stilwell et al., 2024).

These proactive school safety approaches have more research focused on efficacy, including expanded school-based mental health services, mental health first aid, social emotional learning, and school climate focused efforts (Astor et al., 2010). Positive Behavioural Interventions and Supports (PBIS), for example, have been shown to reduce student aggression and enhance emotional regulation (Bradshaw et al., 2012), while trauma-informed school programs help build resilience in students exposed to violence, decreasing their likelihood of carrying weapons (Jaycox et al., 2002). Social emotional learning programs foster emotional intelligence, peer connection, and conflict resolution skills, thereby mitigating risk factors associated with weapon carriage (Durlak et al., 2011). By addressing student social and emotional needs, these programs can enhance positive school climate, strengthening connections between students, teachers, and families. Further, programming could promote belonging and decrease feelings of isolation, which can improve school safety and prevent weapon carriage. These prevention programs could help students develop skills necessary to manage conflict and problem-solve in non-aggressive ways. Programs designed to reduce bullying behaviours, address isolation, and build student engagement in schools can help create the social context where youth do not feel a need to bring weapons to school to demonstrate strength, protect themselves, or retaliate.With school safety efforts growing in prevalence, it is increasingly imperative to develop evidence-based strategies to understand root causes of violence and mitigate future occurrences. The absence of empirical, peer-reviewed evidence on weapon detection systems to prevent weapon carriage in K-12 schools suggests two vital directions for school safety. First, we need to invest in evaluation research utilising comparison group designs to determine what physical environment strategies work to make schools safer without also increasing anxiety among the school community. It is, however, worth noting that conducting rigorous evaluation research in school safety, especially though experimental or quasi-experimental designs, may pose significant challenges. Randomised control trials may be neither feasible nor ethical in school settings, particularly when it involves withholding potentially protective measures from a group of students. Moreover, schools differ widely in their needs, contexts, and levels of existing infrastructure, further complicating the ability to draw generalisable conclusions. These realities underscore the importance of developing innovative, context-sensitive research methodologies that balance rigour with ethical and practical constraints.Second, we need to invest in strategies that have an evidence base and continue to improve their implementation. These strategies focus on the social and early detection environments of schools and focus on precursors of school violence in its many forms (e.g. bullying, isolating youth) that help prevent weapon carriage in schools. In addition to expanding the use of such approaches, there is a critical need to develop evidence-based best practice guidelines that are both effective and feasible for implementation within school settings; guidelines that seek to prevent violence proactively, before it escalates.

Third, relying solely on physical security approaches may not be the best practice, particularly given research indicating that multifaceted, comprehensive approaches are more effective in addressing school safety (Bradshaw et al., 2021; Stilwell et al., 2024). Effective prevention strategies should integrate environmental, behavioural, and relational components, acknowledging the complexity of the factors that contribute to school violence. Finally, the limited number of firearm-specific prevention programs in school contexts may reflect a broader understanding within the field that effective firearm violence prevention requires upstream interventions. Many experts recognise the importance of addressing underlying risk factors and systemic contributors to violence, such as access to firearms, community-level risk, and student mental health, rather than focusing narrowly on reactive, in-school firearm detection efforts. Ultimately, keeping schools safe requires a comprehensive approach that integrates both preventive and responsive strategies, grounded in evidence and informed by the broader social context in which schools operate.

## Conclusion

This scoping review identified a critical gap in the empirical literature on interventions aimed at preventing weapon carriage in K–12 schools. Out of a broad pool of literature, only two studies met the inclusion criteria, both focusing specifically on weapon carriage outcomes: one on random metal detector searches and the other on the Say Something Anonymous Reporting System (SS-ARS). While both showed some potential, methodological limitations in study design, implementation, and outcome measurement prevent strong causal claims.

The scarcity of such studies may reflect a broader understanding among school safety researchers that firearm carriage is merely the visible ‘tip of the iceberg’ in the complex landscape of school violence prevention, underscoring the importance of addressing the social, emotional, and environmental conditions that lie beneath the surface. Despite the potential utility of target-hardening and reporting systems, the scarcity of empirical research focused specifically on preventing weapon carriage highlights a broader evidence gap across all school-based safety interventions. Rather than concluding that only mental health or climate-related interventions are lacking, this review underscores the need for rigorous evaluations of *any* strategy designed to reduce firearm or weapon carriage among students. The minimal number of studies meeting inclusion criteria suggests that the field has yet to systematically test whether commonly implemented practices, ranging from physical security to social–emotional learning initiatives, actually prevent students from bringing weapons onto school grounds.

While we discuss broader research indicating that positive school climates and mental health supports are protective against youth violence, the current evidence does not directly demonstrate their effect on weapon carriage. These approaches should therefore be regarded as theoretically promising avenues for future investigation rather than as empirically validated solutions for this outcome. Continued evaluation of both structural and psychosocial interventions, using robust comparison group designs, is essential to advance the field and guide policy with greater confidence.

While this review presents an important contribution to the literature, it is not without limitation. First, our inclusion criteria were narrowly focused on studies explicitly measuring weapon carriage outcomes, which may have excluded relevant interventions with indirect effects on this behaviour. Second, the small number of eligible studies limits the generalisability of findings and precludes meta-analysis. Finally, the exclusion of grey literature may have limited the comprehensiveness of the evidence base, particularly in capturing strategies that address both physical safety and emotional well-being. Future research should systematically consider broader inclusion criteria and explore diverse sources to capture a wider range of relevant studies. For instance, expanding the scope to include interventions addressing any firearm-related behaviour or offence committed by students, such as possession, threats, display, or firearm-related disciplinary incidents, could yield a more comprehensive evidence base while maintaining conceptual relevance and reflecting the full spectrum of school safety approaches, including those engaging with deeper social and emotional issues.

To inform future decision-making, we recommend prioritising investment in evidence-informed upstream approaches that foster connectedness, equity, and emotional well-being within schools, and funding rigorous evaluations of existing physical security strategies to assess their direct and indirect impacts on weapon carriage, student behaviour, and school climate. Data on implementation fidelity, student perceptions, unintended consequences, and cost-effectiveness are essential to guide future policy and practice. Ultimately, creating safer school environments requires moving beyond reactive security measures toward integrated strategies that address the root causes of fear, disconnection, and violence among youth.This review contributes to the literature by highlighting the scarcity of rigorous, empirical evaluations of school-based weapon prevention strategies and advocating for a shift in focus toward integrated, evidence-informed practices. This work lays a foundation for future research and policy innovation to create safer, more inclusive learning environments by emphasising the importance of social and psychological dimensions of school safety.

## Data Availability

The data presented in this paper are available in the results section, Appendix A provides the PRISMA-ScR checklist, and Appendix B displays search terms used for the review.

## Appendices

### Appendix A

**Table A1.** Preferred reporting items for systematic reviews and meta-analyses extension for scoping reviews (PRISMA-ScR) checklist.

Section	Item	PRISMA-ScR checklist item	Reported on page #
**Title**			
Title	1	Identify the report as a scoping review.	1
Abstract			
Structured summary	2	Provide a structured summary that includes (as applicable): background, objectives, eligibility criteria, sources of evidence, charting methods, results, and conclusions that relate to the review questions and objectives.	2
**Introduction**			
Rationale	3	Describe the rationale for the review in the context of what is already known. Explain why the review questions/objectives lend themselves to a scoping review approach.	10
Objectives	4	Provide an explicit statement of the questions and objectives being addressed with reference to their key elements (e.g. population or participants, concepts, and context) or other relevant key elements used to conceptualise the review questions and/or objectives.	10
**Methods**			
Protocol and registration	5	Indicate whether a review protocol exists; state if and where it can be accessed (e.g. a Web address); and if available, provide registration information, including the registration number.	10
Eligibility criteria	6	Specify characteristics of the sources of evidence used as eligibility criteria (e.g. years considered, language, and publication status), and provide a rationale.	10−11
Information sources^*^	7	Describe all information sources in the search (e.g. databases with dates of coverage and contact with authors to identify additional sources), as well as the date the most recent search was executed.	10
Search	8	Present the full electronic search strategy for at least 1 database, including any limits used, such that it could be repeated.	Appendix B
Selection of sources of evidence^†^	9	State the process for selecting sources of evidence (i.e. screening and eligibility) included in the scoping review.	11−12
Data charting process^‡^	10	Describe the methods of charting data from the included sources of evidence (e.g. calibrated forms or forms that have been tested by the team before their use, and whether data charting was done independently or in duplicate) and any processes for obtaining and confirming data from investigators.	−
Data items	11	List and define all variables for which data were sought and any assumptions and simplifications made.	12
Critical appraisal of individual sources of evidence^§^	12	If done, provide a rationale for conducting a critical appraisal of included sources of evidence; describe the methods used and how this information was used in any data synthesis (if appropriate).	11
Synthesis of results	13	Describe the methods of handling and summarising the data that were charted.	12
**Results**			
Selection of sources of evidence	14	Give numbers of sources of evidence screened, assessed for eligibility, and included in the review, with reasons for exclusions at each stage, ideally using a flow diagram.	12−13 & Figure 1
Characteristics of sources of evidence	15	For each source of evidence, present characteristics for which data were charted and provide the citations.	13-14
Critical appraisal within sources of evidence	16	If done, present data on critical appraisal of included sources of evidence (see item 12).	−
Results of individual sources of evidence	17	For each included source of evidence, present the relevant data that were charted that relate to the review questions and objectives.	13−14
Synthesis of results	18	Summarise and/or present the charting results as they relate to the review questions and objectives.	13
**Discussion**			
Summary of evidence	19	Summarise the main results (including an overview of concepts, themes, and types of evidence available), link to the review questions and objectives, and consider the relevance to key groups.	14
Limitations	20	Discuss the limitations of the scoping review process.	19−20
Conclusions	21	Provide a general interpretation of the results with respect to the review questions and objectives, as well as potential implications and/or next steps.	18−21
**Funding**			
Funding	22	Describe sources of funding for the included sources of evidence, as well as sources of funding for the scoping review. Describe the role of the funders of the scoping review.	22

JBI = Joanna Briggs Institute; PRISMA-ScR = Preferred Reporting Items for Systematic reviews and Meta-Analyses extension for Scoping Reviews.

^*^ Where *sources of evidence* (see second footnote) are compiled from, such as bibliographic databases, social media platforms, and Web sites.

^†^ A more inclusive/heterogeneous term used to account for the different types of evidence or data sources (e.g. quantitative and/or qualitative research, expert opinion, and policy documents) that may be eligible in a scoping review as opposed to only studies. This is not to be confused with information sources (see first footnote).

^‡^ The frameworks by Arksey and O’Malley (6) and Levac and colleagues (7) and the JBI guidance (4, 5) refer to the process of data extraction in a scoping review as data charting.

^§^ The process of systematically examining research evidence to assess its validity, results, and relevance before using it to inform a decision. This term is used for items 12 and 19 instead of ‘risk of bias’ (which is more applicable to systematic reviews of interventions) to include and acknowledge the various sources of evidence that may be used in a scoping review (e.g. quantitative and/or qualitative research, expert opinion, and policy document).

*From:* Tricco AC, Lillie E, Zarin W, O'Brien KK, Colquhoun H, Levac D, et al. PRISMA Extension for Scoping Reviews (PRISMAScR): Checklist and Explanation. Ann Intern Med. 2018;169:467–473. doi: 10.7326/M18-0850.

### Appendix B

***Search Syntax***

**PsycInfo** (Ebsco)

DE ‘Firearms’ OR DE ‘Gun Control Laws’ OR DE ‘Gun Violence’ OR (TI firearm* OR AB firearm*) OR (TI gun OR AB gun) OR (TI guns OR AB guns) OR (TI rifle OR AB rifle) OR (TI rifles OR AB rifles) OR (TI pistol* OR AB pistol*) OR (TI revolver* OR AB revolver*) OR (TI semi-automatic* OR AB semi-automatic*) OR (TI shotgun* OR AB shotgun*) OR (TI ammunition OR AB ammunition) OR (TI handgun* OR AB handgun*) OR (TI gunshot* OR AB gunshot*) OR (TI shooting* OR AB shooting*) OR (TI ‘small arms’ OR AB ‘small arms’).

**AND**

(TI carriage OR AB carriage) OR (TI carrying OR AB carrying) OR (TI carry OR AB carry) OR (TI ownership OR AB ownership) OR (TI own OR AB own) OR (TI owns OR AB owns) OR (TI owning OR AB owning) OR (TI bring OR AB bring) OR (TI bringing OR AB bringing) OR (TI possess* OR AB possess*).

**AND**

DE ‘Schools’ OR DE ‘Boarding Schools’ OR DE ‘Elementary Schools’ OR DE ‘High Schools’ OR DE ‘Junior High Schools’ OR DE ‘Kindergartens’ OR DE ‘Middle Schools’ OR DE ‘Students’ OR DE ‘Elementary School Students’ OR DE ‘Intermediate School Students’ OR DE ‘Primary School Students’ OR DE ‘High School Students’ OR DE ‘Junior High School Students’ OR DE ‘Kindergarten Students’ OR DE ‘Teachers’ OR DE ‘Elementary School Teachers’ OR DE ‘High School Teachers’ OR DE ‘Junior High School Teachers’ OR DE ‘Middle School Teachers’ OR DE ‘Special Education Teachers’ OR (TI school* OR AB school*) OR (TI school-based OR AB school-based) OR (TI student* OR AB student*) OR (TI kindergarten* OR AB kindergarten*) OR (TI pupil* OR AB pupil*) OR (TI teacher* OR AB teacher*).
